# Multicolor Colormetric Biosensor for the Determination of Glucose based on the Etching of Gold Nanorods

**DOI:** 10.1038/srep37879

**Published:** 2016-11-25

**Authors:** Yue Lin, Mengmeng Zhao, Yajuan Guo, Xiaoming Ma, Fang Luo, Longhua Guo, Bin Qiu, Guonan Chen, Zhenyu Lin

**Affiliations:** 1 MOE Key Laboratory of Analysis and Detection for Food Safety, Fujian Provincial Key Laboratory of Analysis and Detection Technology for Food Safety, Institute of Nanomedicine and Nanobiosensing, College of Chemistry, Fuzhou University, Fuzhou, Fujian 350116, China; 2 College of Biological Science and Technology, Fuzhou University, Fuzhou, Fujian 350116, China

## Abstract

In this work, 3,3′,5,5′-tetramethylbenzidine(II) (TMB^2+^), derived from H_2_O_2_-horseradish peroxidase (HRP)-3,3′,5,5′-tetramethylbenzidine (H_2_O_2_-HRP-TMB) reaction system, was used to etch AuNRs to generate different colors of solution. Many enzyme reactions are involved in the production of H_2_O_2_ (e.g., glucose can react with the dissolved oxygen in the presence of glucose oxidase (GOx) to produce H_2_O_2_). Given this information, a simple visual biosensor was developed in this study, with glucose as the example target. The detection range of the proposed system varied with the experimental conditions, such as the concentration of GOx and HRP, and enzymatic reaction time. Under the optimized conditions, the longitudinal shift of localized surface plasmon resonances (LSPR) had a linear correlation with the glucose concentration in the range of 0.1~1.0 mM. Meanwhile, the solution displayed a specific color in response to the glucose concentration, thus enabling the visual quantitative detection of glucose at a glance. Compared with the traditional monochromic colorimetry, this multicolor glucose sensor generates various vivid colors, which can be easily distinguished by naked eyes without any sophisticated instrument. Notably, the proposed method has been successfully applied to detect glucose in serum samples with satisfied results.

The solution of noble metals is normally colored in view of their fascinating size-dependent properties. The color is mainly controlled by their shape, size, and composition, resulting in great wide application prospect in electronics, catalysis, optics, and biosensing^1^,^2^. Among these noble metals, gold nanoparticles (AuNPs) exhibit strong localized surface plasmon resonances (LSPRs) within the visible or near-IR, making them broadly used in colorimetric chernosensor^3^,^4^. Gold nanorods (AuNRs) possess a typical plasmonic one-dimensional nanostructure with two separate surface plasmon resonances (SPR) bands corresponding to transverse and longitudinal peak, respectively, the longitudinal peak is sensitive to their aspect ratio. Different optical signals can be acquired in a wide range of wavelength by simply adjusting the aspect ratio^5^. Based on these characters, AuNRs have gained increasing attention and how to change their aspect ratio becomes a problem worthy of exploring in various application fields. To date, many post-synthetic morphological methods have been proposed for modifying AuNRs such as transverse overgrowth^6^, shorting^7^, and lateral etching^8^. A promising detection method based on the etching of AuNRs by H_2_O_2_ has been recently reported^9^,^10^. Vivid color changes were presented. However, it still faced with some disadvantages. For example, several hours are required to proceed the oxidation of AuNRs with high concentration of H_2_O_2_ and specific pH, making it possess biological destructiveness and hence difficult to be applied in biological detection systems.

To significantly improve the performance of the sensing systems, aplenty of enzymes and metal ions have been employed to shorten the detection time due to their catalyticproperty. Numerous plasmonic sensors and other similar ones have been developed and applied to detect diverse targets other than H_2_O_2_, such as Cu^2+^, Fe^3+^, CN^−^, NO_2_^−^, Cl^−^ and Hg^+^ ions^11^,^12^,^13^,^14^,^15^,^16^,^17^. Methods for Cu^2+^ detection was proposed via the etching of AuNRs by H_2_O_2_ because Cu^2+^ can catalyze H_2_O_2_-AuNRs etching system^18^,^19^. A promising strategy was reported to detect Fe^3+^ through selective etching of Au NRs at room temperature^13^. A method was developed for blood glucose detection based on enzymatic etching of AuNRs by horseradish peroxidase (HRP)^20^. High concentration of HRP, however, is needed in this system, which extremely limits the reaction conditions. Based on the similar principle, a plasmonic monitor was proposed for blood glucose^21^. Nevertheless all these chromogenic processes in sensors cannot be terminated or kept stable at a fixed time range (for easily discrimination). Furthermore, the color change cannot be easily distinguished by naked eyes.

HRP-H_2_O_2_-3,3′,5,5′-tetramethylbenzidine (TMB) system has been used to quantify H_2_O_2_ frequently and this principle has been coupled with enzymatic reaction (which can produce H_2_O_2_) to detect diverse targets with high selectivity. In this study, the product of H_2_O_2_-HRP-TMB-HCl catalyzed oxidation system, 3,3′,5,5′-tetramethylbenzidine(II) (TMB^2+^), quantitatively etched AuNRs to produce different vivid color solutions^22^, meanwhile, the addition of HCl can inactivate effectively HRP to keep the oxidizing reaction stable. Because the TMB^2+^ has direct relationship with H_2_O_2_ and H_2_O_2_ can be generated from glucose-GOx reaction system, glucose was chosen as the example. A novel multicolor glucose sensor utilizing TMB^2+^-AuNRs as chromogenic substrate is presented to realize the visual quantitative detection of glucose by naked eyes. The multicolor glucose sensor displays advantages of speediness, simplicity, visualization as well as low cost, enabling this system to be a potentially powerful tool for the investigation of blood glucose in clinical examination, especially for some poor areas lacking expert medical facilities.

## Methods

### Materials and Instruments

TMB, HRP, HAuCl_4_ and glucose were acquired from Aladdin (Shanghai, China), while the cetyltrimethyl ammonium bromide (CTAB) was obtained from J&K Chemical Technology (Beijing, China) and the ascorbic acid (AA) was supplied by Fu Chen Chemistry (Tianjin, China). Amino acids, NaBH_4_, AgNO_3_, and H_2_O_2_ were purchased from Sinopharm (Shanghai, China). GOx was purchased from Sangon (Shanghai, China). All used water was ultrapure water (18.2 > MΩ•cm) from Direct-Q3 UV system (Millipore). Ultraviolet-visible (UV-Vis) absorption spectra were recorded by Multiskan spectrum microplate spectrophotometer (Thermo, USA) and UV-Vis absorption spectroscopy (1102UV-Vis spectrophotometer, Techcomp, China).

### Preparation of Gold Nanorods

According to the previously reported literature^7^, AuNRs were synthesized with slight modification. Briefly, 0.25 mL of HAuCl_4_ (0.01 M) mixed with 5 mL of CTAB (0.2 M) in a 20 mL glass bottle under water bath (30 °C). 0.6 mL of NaBH_4_ (0.01 M) freshly prepared by ice distilled water was rapidly added to the glass bottle, followed by vigorous inversion (1200 r/min) for 5 min. The color of the liquid changing from dark yellow to brown indicated the formation of seed solution. Keep it stationary at room temperature for at least 10 min. Subsequently, 100 mL of CTAB (0.2 M) was added to 1.25 mL of AgNO_3_ (0.01 M) in 250 mL florence flask with vigorous stirring (700 r/min) for 2 min, and then 10 mL of HAuCl_4_ (0.01 M) was introduced to the mixture. The final mixture was continuously stirred for 2 min. 11 mL of ascorbic acid (0.01 M, AA) was slowly dripped into the resulting mixture until the color turned from pale yellow to colourless. Finally, 0.4 mL of seed solution was quickly added to resultant mixture with sufficient oscillation for 20 sec. Keep it at 30 °C in a water bath for at least 3 h to ensure full growth of AuNRs. The CTAB on the surface of AuNRs was removed by centrifugation and the resulting concentrated AuNRs solution was kept in a 20 mL brown glass bottle to extend the shelf-life.

### Visual Detection of Glucose

20 mL of CTAB solution (0.2 M) initially mixed with concentrated AuNRs solution at a volume ratio of 4:1. Different concentrations of glucose (50 μL) were added to 96-well plates at room temperature, and then 400 μL of GOx (0.1 mg/mL), 400 μL of HRP (4 μg/mL) and 1 mL of TMB liquid substrate were added to the 4 mL centrifuge tube with sufficient mixing. Subsequently, 90 μL of obtained solution was added to glucose in the 96-well plates, the light blue slowly appeared and deepened over time. After enzymatic reaction (20 min), 10 μL of HCl (5 M) was introduced to the mixture to end the reaction. The color quickly turned to yellow, suggesting TMB^2+^ was produced^23^. 100 μL of AuNRs-CTAB mixture was eventually added to the 96-well plates. After sufficient reaction (within 60 s), the absorption spectra ranging from 300 to 900 nm were recorded using the Multiskan spectrum microplate spectrophotometer.

### Detection of Glucose in Serum Samples

Different serum samples were diluted with PBS (0.01 M, pH7.4) at a volume ratio of 1:9. Then 50 μL of diluent solution was added to the 96-well plates. In parallel, 50 μL of distilled water was added to the other well and the next procedure was the same as the abovementioned process for glucose sensing.

## Results and Discussion

### Principle of the Proposed Multicolor Glucose Senor

**Figure 1:**
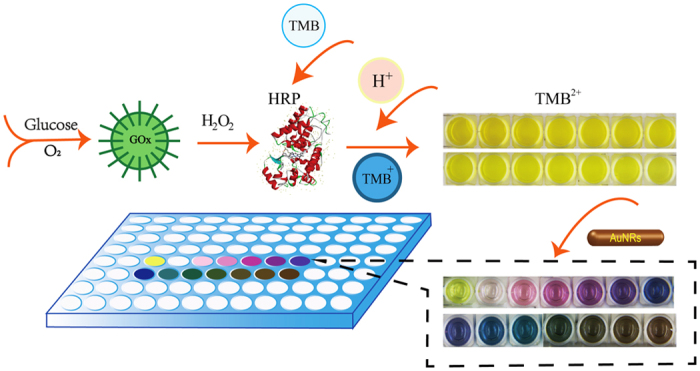
Presentation of muticolor glucose sensor by the oxidation of Au NRs.

Figure 1 schematically depicts the principle of the proposed multicolor sensor. With the assistant of GOx, glucose reacts with the dissolved oxygen to produce H_2_O_2_. TMB^0^ is then oxidized to blue TMB^+^ by H_2_O_2_ in the presence of HRP. After the addition of HCl, HRP is inactivated and the yellow TMB^2+^ is produced. However, the TMB^2+^ just presents various shades of yellow, which is difficult for discrimination of human eyes, Then the mixture solution of AuNRs and CTAB is added to the TMB^2+^ solution with sufficient mixing. AuNRs can react with TMB^2+^ to produce TMB^0^, and meanwhile Au was oxidized into Au(I). This oxidation process selectively takes place at the ends of AuNRs^7^. Thus, the visual glucose detection can be achieved based on the etching of AuNRs. Different levels of etching and various colors of the solution respond directly to different concentrations of glucose. The final obtained solution is rich-colored, several colors such as pink, purple, blue, green and grey at different glucose concentrations can be distinguished at a glance. When the AuNRs are completely oxidized, only TMB^2+^ exists and the color changes to yellow. Based on these phenomena, a pretty simple multicolor biosensor was developed for the determination of glucose.

**Figure 2:**
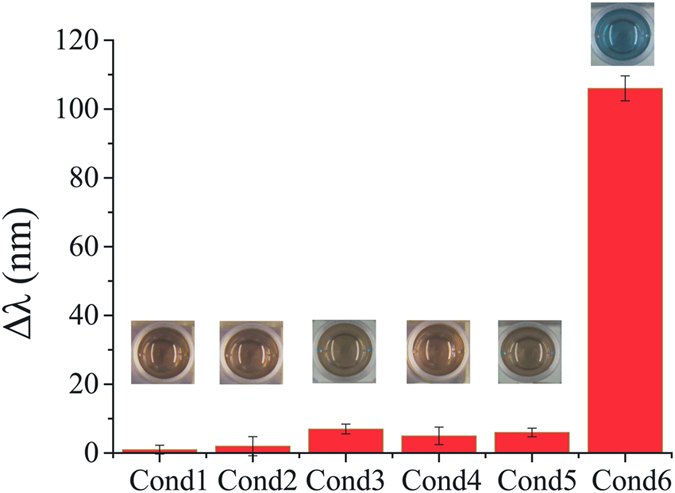
The LSPR shift of Au NRs under different conditions. (Cond 1: Glucose + GOx + HRP + AuNRs; Cond 2: Glucose + HRP + TMB + HCl + AuNRs; Cond 3: GOx + HRP + TMB + HCl + AuNRs; Cond 4: Glucose + GOx + TMB + HCl + AuNRs; Cond 5: Glucose + GOx + HRP + HCl + AuNRs; Cond 6: Glucose + GOx + HRP + TMB + HCl + AuNRs). The insets show the solution color of the corresponding conditions.

**Figure 3:**
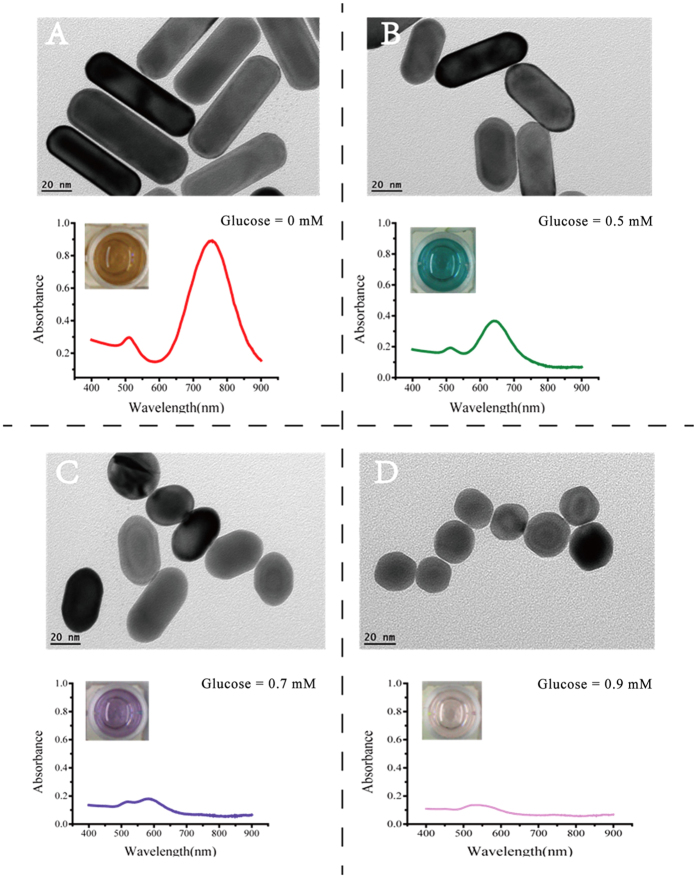
The UV-Vis absorption spectra and TEM images of AuNRs before and after etching with different concentrations of TMB^2+^. The insets show the colors of the corresponding solutions.

Control experiments were carried out to demonstrate the feasibility of the proposed system. Figure 2 shows the performance of the system under different conditions. Under the several conditions without the addition of HCl and TMB, GOx, glucose, HRP, or TMB, respectively, no color change was observed, indicating the unsuccessful etching of AuNRs. Whereas the color of the solution changed quickly when all reagents were simultaneously added, indicating the successful etching of AuNRs. These results confirm the feasibility of principle. Figure 3 Adisplays the TEM image and the corresponding SPR absorption spectrum of AuNRs without addition of TMB^2+^. While Fig. 3B,C and D show the TEM images and the SPR absorption spectra of AuNRs with addition of different amounts of glucose and the rest of reagent (used to produce different quantity of TMB^2+^). With the increase of glucose concentration, the length of AuNRs decreased gradually, at the same time, the longitudinal LSPR shifted blue and the solution displayed different colors. Notably, these results also verify the feasibility of principle.

### Optimization of the Reaction Conditions

The concentration of GOx and HRP, and enzymatic reaction time have a great influence on the performance of the proposed biosensor because they affect the amount of H_2_O_2_ produced. Given that the normal blood glucose in the human serum ranges from 3.9 to 7.8 mM, to reduce the potential interference, the sample was diluted for 10 times before examination. To fit the real glucose level in human serum, the sensing range was controlled from 0.1 to 1.0 mM. Excessive amount of GOx (0.01 mg/mL) was added and the enzymatic reaction was fixed at 20 min for easy detection.

**Figure 4:**
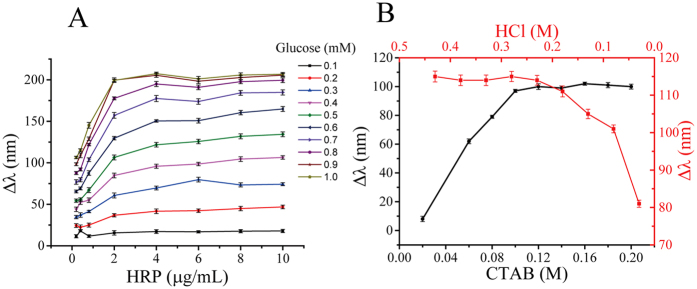
(**A**) The LSPR shift of AuNRs with various concentrations of HRP (initial concentrations: 0.4, 0.6, 0.8, 2.0, 4.0, 6.0 8.0, 10.0 μg/mL, respectively); (**B**) The LSPR shift of AuNRs with various concentrations of CTAB (initial concentrations: 0.03, 0.06, 0.10, 0.12, 0.13, 0.14, 0.16, 0.18, 0.20 M, respectively) and HCl (initial concentrations: 0.03, 0.08, 0.13, 0.18, 0.23, 0.28, 0.33, 0.38, 0.43 M, respectively).

The concentration of HRP was optimized firstly. As shown in Fig. 4A, the longitudinal peak shift of AuNRs increased with the augment of HRP concentration at different levels of glucose, and then reached a plateau when higher than 4 μg/mL. So 4 μg/mL of HRP was chosen as the optimal condition. CTAB also plays an important role in the proposed multicolor senor system. In the absence of CTAB, the standard electrode potential of AuBr^2−^/Au (0.93 V vs NHE) (E^Θ^) was lower than TMB^2+^/TMB (0.741 V vs NHE), preventing the oxidation of AuNRs by TMB^2+^. Upon the addition of CTAB, the reduction potential of AuBr^2−^/Au reduced via forming AuBr^2−^ − (CTA)^2+^/Au (E^Θ^ < 0.2 V vs NHE), facilitating the oxidation of AuNRs^24^. The concentration of CTABwas optimized as well, and the result showed that as CTAB concentration rose, the longitudinal peak shift of AuNRs first increased rapidly and then kept stable when its concentration exceeded 0.12 M (Fig. 4B). So 0.12 M was chosen as the optimal concentration of CTAB. HCl in this detection is used to inactive HRP to stop the reaction, so it is necessary to explore how the presence of HCl affects the detection results. As shown in Fig. 4B, the longitudinal peak shift of AuNRs was increased with the addition of HCl and then reaches a plateau when the concentration of HCl was over 0.23 M. So, 0.23 M was chosen as the optimal concentration.

### Calibration Curve for Glucose Determination

**Figure 5:**
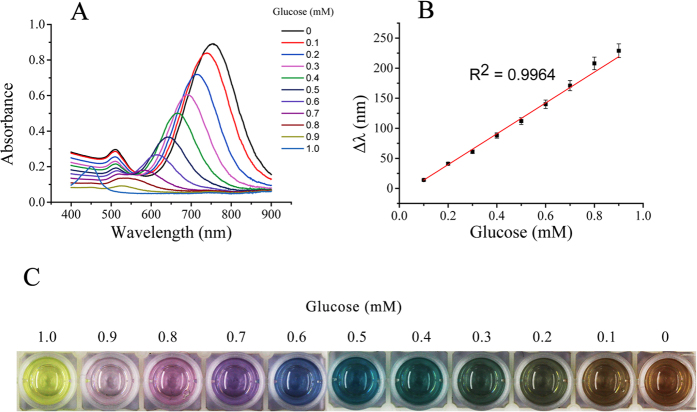
(**A**) The UV-Vis spectra change of AuNRs in the oxidation etching process with different concentrations of glucose; (**B**) The LSPR shift of AuNRs as a function of glucose concentration; (**C**) Color change of the plasmonic sensor with the decrease of glucose concentrations.

**Figure 6:**
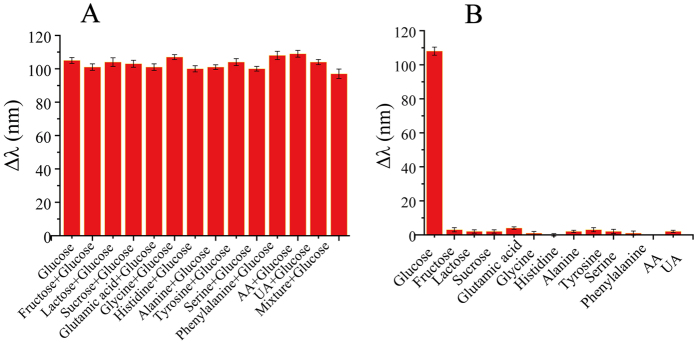
Selectivity tests of the multicolor glucose sensor to normal interference in traditional glucose assay. ∆λ: the LSPR shift of the AuNRs in response to (**A**) 0.5 mM glucose, 1 mM Fructose, 1 mM Lactose, 1 mM sucrose, 30 mM Glutamic acid, 10 mM Glycine, 10 mM Histidine, 30 mM Alanine, 30 mM Tyrosine, 30 mM Serine, 6 mM Phenylalanine, 1 mMAA and 1 mM UA and (**B**) the mixture of 0.5 mM glucose and 1 mM Fructose, 1 mM Lactose, 1 mM sucrose, 30 mM Glutamic acid, 10 mM Glycine, 10 mM Histidine, 30 mM Alanine, 30 mM Tyrosine, 30 mM Serine, 6 mM Phenylalanine, 1 mM AA and 1 mM UA.

Different concentrations of glucose were added to the homogeneous solution containing HRP, TMB, GOx and HCl under the optimized conditions. A gradual blue-shift of LSPR was observed in Fig. 5A. As presented in Fig. 5B, the longitudinal LSPR shift had a linear correlation with the concentration of glucose in the range between 0.1 and 0.9 mM. Furthermore, different colors appeared with different concentrations of glucose (Fig. 5C). Accordingly, the concentration of glucose can be well determined via vivid color change with high sensitivity, which can be observed by naked eyes at a glance. Interference studies were done to explore the specific detection of glucose in human serum using this sensor, including investigation of most commonly existing substances in human serum such as fructose, sucrose, ascorbic acid (AA), uric acid (UA) and a series of amino acids (glutamic acid, glycine, histidine, alanine, tyrosine, serine, and phentlalanine). Given the approximate amount of these samples in serum and quantity demand of real samples, different concentrations of abovementioned interferences were separately added to the detection system with 0.5 mM of glucose. The absorbance results barely changed, indicating these interferences hardly affect the glucose sensing (Fig. 6A). Furthermore, Fig. 6B also demonstrated the excellent sensitivity of the assay using the same interferences. This is probably due to the specificity between GOx and glucose. Thus this result demonstrates that the proposed multicolor glucose sensor owns superior selectivity.

### Visual Detection of Glucose in Serum Samples

**Figure 7:**
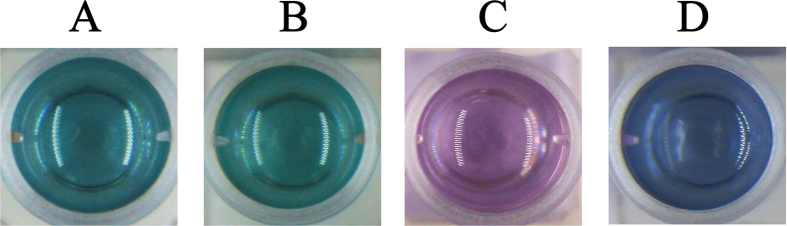
The solution color of AuNRs for the corresponding human serum samples in Table 1.

Serum samples obtained from the volunteers were firstly diluted 10 times. The results reveal that the solution colors present green, blue and purple (Fig. 7). According to the above standard colorimetric figure, the concentration of glucose in Sample A was inferred to be between 4.0 and 5.0 mM, which was close to the calculated value of 4.8 mM, and the Sample B to D obeyed the same rule (Table 1). When the solution color turn to purple, it means the blood-sugar level is out of normal value (approximately 7.0 mM). These results indicate that our proposed multicolor glucose sensor has great potential application in the detection of real samples.

## Conclusions

In conclusion, a multicolor glucose sensor has been proposed due to the special optical property of AuNRs and the etching of AuNRs by TMB^2+^. As the glucose concentration increased, the LSPR band of AuNRs had a blue-shift and the solution showed the vivid colors from the reddish brown, gray, green, blue, purple, pink to yellow. The visual detection for glucose is achieved by naked eyes with the advantages of speediness, simplicity, and low cost.

## Additional Information

**How to cite this article**: Lin, Y. *et al*. Multicolor Colormetric Biosensor for the Determination of Glucose based on the Etching of Gold Nanorods. *Sci. Rep.* **6**, 37879; doi: 10.1038/srep37879 (2016).

**Publisher’s note:** Springer Nature remains neutral with regard to jurisdictional claims in published maps and institutional affiliations.

## Figures and Tables

**Table 1:** Visual detection for glucose in human serum.

Sample	Glucose (mM)	
	Calculated value	This method
A	4.8	4.0~5.0
B	4.5	4.0~5.0
C	7.2	7.0~8.0
D	5.7	5.0~6.0
